# Distinct neuron populations for simple and compound calls in the primary auditory cortex of awake marmosets

**DOI:** 10.1093/nsr/nwab126

**Published:** 2021-07-12

**Authors:** Huan-huan Zeng, Jun-feng Huang, Jun-ru Li, Zhiming Shen, Neng Gong, Yun-qing Wen, Liping Wang, Mu-ming Poo

**Affiliations:** Center for Excellence in Brain Science and Intelligence Technology, Institute of Neuroscience, State Key Laboratory of Neuroscience, CAS Key Laboratory of Primate Neurobiology, Chinese Academy of Sciences, Shanghai 200031, China; Shanghai Center for Brain Science and Brain-Inspired Intelligence Technology, Shanghai 200031, China; Center for Excellence in Brain Science and Intelligence Technology, Institute of Neuroscience, State Key Laboratory of Neuroscience, CAS Key Laboratory of Primate Neurobiology, Chinese Academy of Sciences, Shanghai 200031, China; University of Chinese Academy of Sciences, Beijing 100086, China; Shanghai Center for Brain Science and Brain-Inspired Intelligence Technology, Shanghai 200031, China; Center for Excellence in Brain Science and Intelligence Technology, Institute of Neuroscience, State Key Laboratory of Neuroscience, CAS Key Laboratory of Primate Neurobiology, Chinese Academy of Sciences, Shanghai 200031, China; Shanghai Center for Brain Science and Brain-Inspired Intelligence Technology, Shanghai 200031, China; Center for Excellence in Brain Science and Intelligence Technology, Institute of Neuroscience, State Key Laboratory of Neuroscience, CAS Key Laboratory of Primate Neurobiology, Chinese Academy of Sciences, Shanghai 200031, China; Shanghai Center for Brain Science and Brain-Inspired Intelligence Technology, Shanghai 200031, China; Center for Excellence in Brain Science and Intelligence Technology, Institute of Neuroscience, State Key Laboratory of Neuroscience, CAS Key Laboratory of Primate Neurobiology, Chinese Academy of Sciences, Shanghai 200031, China; Shanghai Center for Brain Science and Brain-Inspired Intelligence Technology, Shanghai 200031, China; Center for Excellence in Brain Science and Intelligence Technology, Institute of Neuroscience, State Key Laboratory of Neuroscience, CAS Key Laboratory of Primate Neurobiology, Chinese Academy of Sciences, Shanghai 200031, China; Shanghai Center for Brain Science and Brain-Inspired Intelligence Technology, Shanghai 200031, China; Center for Excellence in Brain Science and Intelligence Technology, Institute of Neuroscience, State Key Laboratory of Neuroscience, CAS Key Laboratory of Primate Neurobiology, Chinese Academy of Sciences, Shanghai 200031, China; Shanghai Center for Brain Science and Brain-Inspired Intelligence Technology, Shanghai 200031, China; Center for Excellence in Brain Science and Intelligence Technology, Institute of Neuroscience, State Key Laboratory of Neuroscience, CAS Key Laboratory of Primate Neurobiology, Chinese Academy of Sciences, Shanghai 200031, China; University of Chinese Academy of Sciences, Beijing 100086, China; Shanghai Center for Brain Science and Brain-Inspired Intelligence Technology, Shanghai 200031, China

**Keywords:** marmoset calls, calcium imaging, awake marmosets, primary auditory cortex, vocal communication

## Abstract

Marmosets are highly social non-human primates that live in families. They exhibit rich vocalization, but the neural basis underlying this complex vocal communication is largely unknown. Here we report the existence of specific neuron populations in marmoset A1 that respond selectively to distinct simple or compound calls made by conspecific marmosets. These neurons were spatially dispersed within A1 but distinct from those responsive to pure tones. Call-selective responses were markedly diminished when individual domains of the call were deleted or the domain sequence was altered, indicating the importance of the global rather than local spectral-temporal properties of the sound. Compound call-selective responses also disappeared when the sequence of the two simple-call components was reversed or their interval was extended beyond 1 s. Light anesthesia largely abolished call-selective responses. Our findings demonstrate extensive inhibitory and facilitatory interactions among call-evoked responses, and provide the basis for further study of circuit mechanisms underlying vocal communication in awake non-human primates.

## INTRODUCTION

Marmosets are considered to be an excellent animal model for studying neural substrates underlying complex vocal communication [1,2]. Previous brain imaging and electrophysiological studies of primate auditory systems have shown that neurons in the rostral temporal lobe show high preference for complex vocal sounds [3–5], whereas neurons in more caudal areas such as the primary auditory cortex (A1) are well-known for their tonotopic properties, with neurons clustered into regions preferring specific frequencies [6,7]. In addition to their frequency preference, A1 neurons are also sensitive to specific spectral-temporal features of the sound, e.g. harmonicity [8], frequency and temporal modulation [9]. Electrophysiological studies in the A1 of anesthetized marmosets have detected neurons that responded selectively to a simple Twitter call [10]. However, it is unclear whether A1 neurons could selectively respond to all natural calls, including both simple calls and compound calls (comprising sequences of simple calls), and whether call-evoked responses were only due to the neurons’ sensitivity towards specific local spectral-temporal features of the sound, or require global temporal organization of various sound components, such as the sequence and interval of simple call components within the compound call. It is thus important to perform simultaneous recordings of the activity from large A1 neuron populations in the same marmoset. Such recordings need to be conducted in the awake state, since anesthesia is known to greatly reduce neuronal activity in the cortex.

In this study, we have achieved two-photon fluorescence imaging of large populations of A1 neurons in un-anesthetized marmosets by acute loading of Ca^2+^-sensitive fluorescent dye Cal-520AM. This method allows rapid labeling of a much larger proportion of neurons than could currently be achieved by genetic expression of GCaMP6. Using this method, we have identified, within conventional tonotopic regions of A1, substantial populations of neurons that respond selectively to different conspecific simple and compound calls but not to pure tones. Further studies focusing on compound call-selective neurons showed that their responses are sensitive to the sequence and interval of simple call components, characteristics of vocal sound processing. These compound call-selective responses were found only for naturally occurring, but not artificially constructed, compound calls, and were completely abolished by light anesthesia. These findings established the existence of substantial call-selective neuron populations in the A1 of awake marmosets, pointing to complex vocal sound processing in the early stage of the auditory system.

## RESULTS

### Two-photon Ca^2+^ imaging of neuronal activity in A1

We simultaneously monitored the activity of a large population of A1 neurons in head-fixed awake common marmosets by fluorescence Ca^2+^ imaging. The A1 area was first identified based on its tonotopic organization, as revealed by imaging intrinsic optical signals in anesthetized marmosets (Supplementary Fig. 1) [6,7]. Synthetic Ca^2+^-sensitive dye Cal-520AM [6] was then loaded into a specific subregion of A1 (sensitive to 2–8 kHz) to label the neurons of layer 2/3 (see Methods). Two-photon Ca^2+^ imaging of neuronal activity in response to various natural calls (Supplementary Fig. 2a and b; Supplementary Video 1) was performed 2 h after dye loading when the marmoset regained wakefulness, and the recording normally lasted for 3 h.

In an alternative approach, we injected a tetracycline (Tet)-activated Adeno Associated Virus (AAV) vector expressing genetically encoded Ca^2+^-indicator GCaMP6f [11] into A1 and performed imaging more than 4 weeks after injection and 3 days after Tet feeding (Supplementary Fig. 2c and d; see Methods; Supplementary Video 2). Although GCaMP6f expression was detectable in a lower proportion of neurons compared to Cal-520AM, this approach allowed repetitive recording from the same neuron populations, showing the stability of call-evoked neuronal responses in the same marmoset over durations up to at least 1 week (Supplementary Fig. 2e and f). Both imaging approaches yielded similar results, and the data were pooled in some analyses.

### Selective responses for conspecific calls

To detect neurons that could respond selectively to the same calls made by conspecific marmosets, we performed two-photon imaging of neuronal Ca^2+^ signals in A1 sub-regions of two marmosets (M_a_ and M_b_) that were acutely loaded with Cal-520AM, and monitored neuronal responses to Phee, Twitter and TrillPhee calls recorded from three other marmosets (M_1_, M_2_, M_3_; three call examples for each call category, 27 calls in total, spectrograms shown in Supplementary Fig. 3a, Supplementary Videos 3–5). Analysis of the spectral-temporal properties of the 27 call samples by principal component analysis and bandwidth Wiener entropy showed a clustered distribution of calls of the same category, despite substantial differences in the duration and spectral-temporal properties among calls made by different marmosets (Supplementary Fig. 3b).

**Figure 1.**
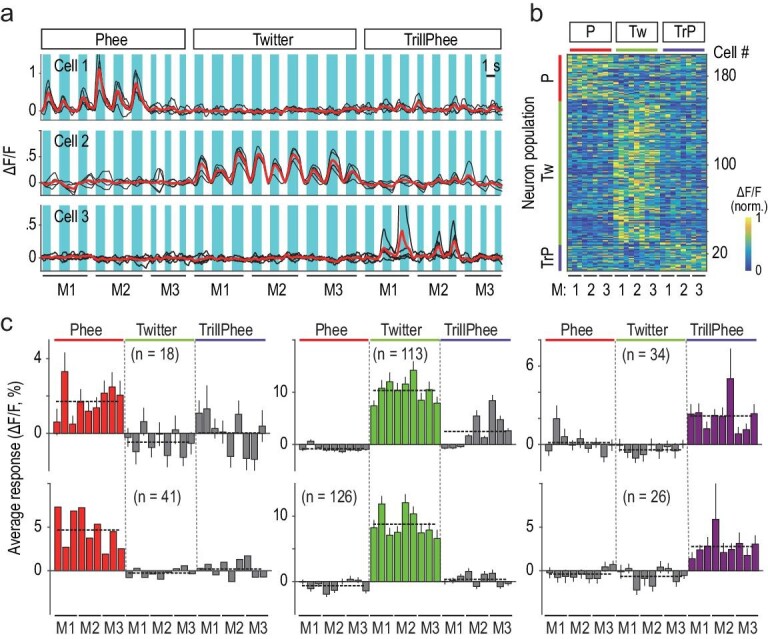
A1 neurons in awake marmosets selectively responded to conspecific calls. (a) Fluorescence changes (ΔF/F) in three example cells (in marmoset M_a_) evoked by 27 conspecific test calls. Note that each cell responded selectively to either Phee, Twitter or TrillPhee calls (n = 3) from three different marmosets (M_1_, M_2_ and M_3_). All stimuli were randomized for presentation. Black traces: single trials (n = 5); red traces: average; shading: call duration. (b) Heat map for all call-selective neurons in one marmoset (M_a_) that was exposed to three call categories as in (a). Each horizontal line depicts the average amplitude of ΔF/F (from five trials), with three representative calls from each marmoset for each call. The cells were sorted into three neuron populations, based on the call that exhibited the highest mean ΔF/F amplitude. The amplitude is coded in color by the scale shown on the right. The numbers at the bottom indicate marmoset identity (M_1_, M_2_ and M_3_). (c) Average response amplitudes of neuron populations that selectively responded to Phee, Twitter and TrillPhee (error bar, Standard Error of Mean (SEM); n = total number of neurons examined). Data were from marmoset M_a_ (bottom) and M_b_ (top) respectively. Dashed horizontal lines: mean response of each neuron population. P = Phee; Tw = Twitter; TrP = TrillPhee.

As illustrated in Fig. 1a, each of the three example neurons from M_a_ showed selective responses to the same category of calls made by two or three different marmosets. We defined neuronal responses to be call-selective when the mean Ca^2+^ fluorescence change (ΔF/F) evoked by a call category (n = 9, three calls from each marmoset) was significantly higher (at a level larger than 5-fold) than those evoked by the two other call categories (*P* < 0.05, ANOVA; see Methods). Average responses (ΔF/F) of all call-selective neurons in marmoset M_a_ evoked by 27 call samples were depicted by the heat map in Fig. 1b. A summary of all data from M_a_ and M_b_ showed consistent selectivity of the same neuron population towards conspecific calls (Fig. 1c).

We have examined whether neurons responding selectively to calls could also respond to variables other than the call type, such as call duration, bandwidth, Wiener entropy, amplitude modulation, mean frequency and caller identity (M_1_, M_2_ or M_3_). A generalized linear model was used to perform multi-variable analysis of call-selective neuron populations identified in the experiment described in Fig. 1. We indeed found that the responses of many neurons were significantly modulated by one or more variables other than the call type (Supplementary Fig. 4, see details in Methods). However, a substantial fraction of neurons (25/193, 13.0%, M_a_; 21/165, 12.7%, M_b_) showed an exclusive selectivity to the call type but not to other variables, indicating the existence of neuron populations in A1 that are purely call-type-selective, without being affected by other acoustic factors and caller identity (Supplementary Fig. 4a, M_a_; Supplementary Fig. 4f, M_b_). Furthermore, using multidimensional scaling to visualize neuronal representations of call-type and non-call-type variables (Supplementary Fig. 4b–e, M_a_; Supplementary Fig. 4g–j, M_b_), the exclusive call-type-selective neurons showed three distinct clusters. No such clustering was observed for neurons selective to non-call-type variables, as shown by the absence of distinct P and TrP neuron clusters. Thus, call-selective neurons consisted of neurons that responded exclusively to distinct call types as well as neurons whose call-selective responses were significantly modulated by acoustic variables and caller identity.

### A1 neuronal responses to four standard calls

**Figure 2.**
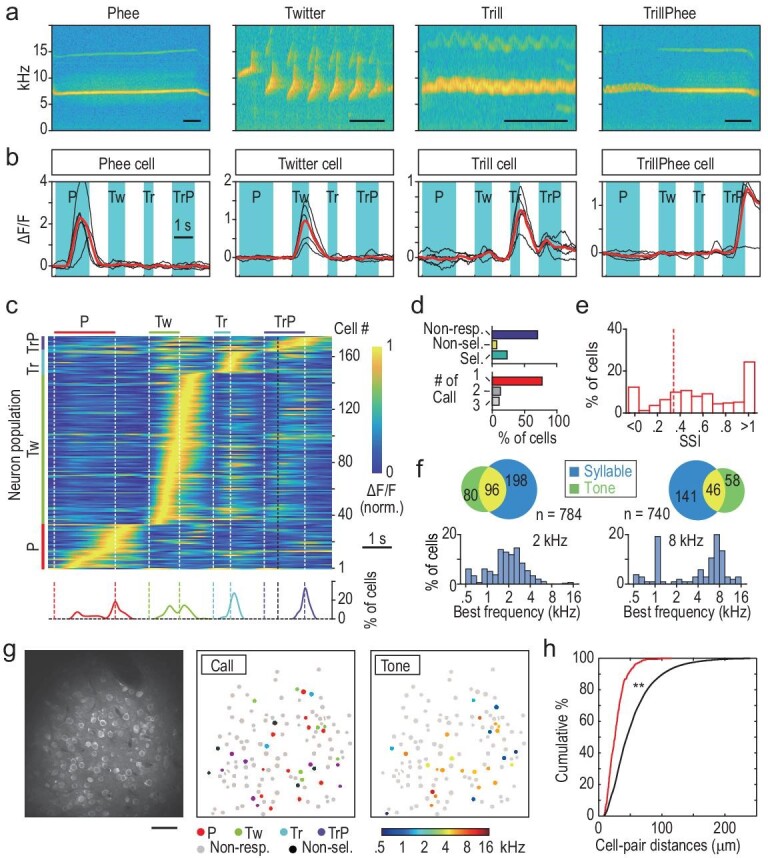
Analysis of call-selective cells in awake marmoset A1. (a) Representative spectrograms of four standard test calls. Bars: 0.2 s. (b) Fluorescence changes (ΔF/F) in four call-selective cells in A1, recorded from marmoset M_c_ that was loaded with Cal-520AM. Black traces: single trials (n = 5); red traces: average; cyan shading: call duration. (c) Heat map for the activity of all call-selective cells in M_c_, with the cells sorted in an order based on the time of peak ΔF/F. White dashed lines: call onset and offset; black dashed line: boundary of Trill-like and Phee-like components of TrillPhee. Bottom: traces depicting percentages of cells that had different peak-response times within each call population. (d) Statistics on call-selective cells recorded from 24 imaging fields in three marmosets (M_a_, M_c_ and M_d_) labeled with Cal-520AM. Top: among all cells recorded (n = 2891), the percentages of cells that were unresponsive, responsive but not call-selective and call-selective. Bottom: the percentages of cells showing call selectivity to one, two or three calls. (e) Call Selective Index (CSI) of all call-selective cells. Red dashed line: CSI = 0.33 (2-fold preference). (f) Top: Venn chart of the number of call-selective neurons and pure-tone responsive neurons, with the overlap representing the number of cells with both types of responses. Bottom: the percentage of pure-tone responsive neurons showing different best frequencies (left, 2-kHz area; right, 8-kHz area). (g) Left: an image of Cal-520AM fluorescence at a recorded region. Bar: 50 μm. Middle: spatial distribution of all cells in the imaging field, with cell response properties coded in colors. Right: tonotopic properties of the imaging field. (h) Cumulative percentage plot of nearest-neighbor distances for cells of the same call selectivity (red line), and for all cells regardless of call selectivity, obtained by bootstrap analysis (black line, see Methods). The difference between two distributions is significant at *P* < 0.001, Kolmogorov-Smirnov test. P = Phee; Tw = Twitter; Tr = Trill; TrP = TrillPhee.

To further investigate the population characteristics and spatial distribution of call-selective neurons in A1, we adopted four of the most common marmoset calls in the standard test set (three simple calls: Phee, Twitter and Trill, and one compound call, TrillPhee; Fig. 2a). Many A1 neurons responded selectively to a specific call (examples in Fig. 2b), and all neurons showing call selectivity were sorted according to the time of the peak ΔF/F signal to obtain the activity profile map, revealing clear call-selective neuron populations within the imaged A1 area of marmoset M_c_ (Fig. 2c). Data for two other marmosets, M_a_ and M_d_, are shown in Supplementary Fig. 5a. Notably, within each neuron population the peak response time of neurons tiled the entire call duration (from hundreds of milliseconds to >1 s), with more neurons reaching peak firing near the end of the call sound (Fig. 2c). As discussed later, this temporal tiling of neuronal responses over the duration of ∼1 s is critical for interval timing in facilitatory and inhibitory interactions among call-evoked responses. The relative sizes of call populations appeared to be different among the three marmosets examined.

TrillPhee is generally viewed as a single discrete call type rather than a combination of a Trill and a Phee, which is evidenced by the observation that the Trill-like and Phee-like components of the TrillPhee showed narrower spectral-temporal bandwidths than those of isolated Trill and Phee (Supplementary Fig. 6). This is consistent with the fact that Phee neurons did not respond to TrillPhee even though it contains the Phee-like component.

Among all A1 neurons examined in three marmosets (M_a_, M_c_ and M_d_) loaded with Cal-520AM, we found that ∼23% (674/2891) showed significantly higher mean response amplitude to one or more calls (*P* < 0.05, ANOVA). A small fraction of them (75/674) exhibited similar mean response amplitudes for two or three calls (*P* > 0.05, *t*-test; Fig. 2d, Supplementary Fig. 5b), and a few showed positive ΔF/F to one call but negative ΔF/F to another (Supplementary Fig. 5c). Among call-selective neurons, Twitter neurons were most common, followed by Phee, TrillPhee and Trill neurons (Supplementary Fig. 5b). Twitter neurons were also the prominent type of call-responsive neurons observed in electrophysiological studies of anesthetized animals [10]. Quantification by Call Selectivity Index (CSI, see Methods) showed that most call-selective neurons exhibited high selectivity (with CSI > 0.33, or a 2-fold difference, Fig. 2e).

### Neuron populations selectively responding to pure tones or calls

The A1 sub-regions chosen for the above experiments had tonotopic preference for either ∼2 or ∼8 kHz, as determined by imaging intrinsic optical signals. Neurons were considered pure-tone-selective based on conventional criteria [6], and all A1 neurons within the imaging field were examined for the responses evoked by pure tones ranging from 0.5 to 16 kHz (five frequency samples per octave). Call-selective neurons were determined by the criteria described above. Our measurements of all Cal-520AM-labeled neurons (n = 784 in M_a_ and 740 in M_c_) showed that only a small percentage of neurons responded selectively to both calls and pure tones (Fig. 2f; 8-kHz area, 6%; 2-kHz area, 12%). Furthermore, the percentage of call neurons was higher than pure-tone neurons in the 8-kHz area (25% vs. 14%), and the opposite was found for the 2-kHz area (22% vs. 38%, Fig. 2f). Moreover, call-selective response amplitudes in the 8-kHz area were slightly larger than in the 2-kHz area (Supplementary Fig. 7), consistent with the observation that most calls exhibited dominant powers around 8 kHz. Further examination of the spatial distribution of different call-selective and pure-tone-selective neurons within the same imaging fields showed that call-selective neurons appeared to be spatially intermingled (Fig. 2g, Supplementary Fig. 8). However, the nearest-neighbor distances for neurons of the same call selectivity were on average smaller than those for neurons randomly sampled from call-selective neuron populations (Fig. 2h, *P* < 0.001, bootstrap analysis), suggesting some spatial clustering of neurons of the same call selectivity.

The call-selective neurons were found to be relatively sparse and dispersed within A1 tonotopic areas, unlike the clustering of face-selective neurons in the inferior temporal cortex. The tendency of closer apposition among neurons with the same call selectivity may reflect intracortical circuit organization underlying call-selective responses. Although the proportion of call-selective neurons in A1 within each imaged field appeared to be relatively low (<10% for Twitter cells), the estimated total A1 neuron population for each call type could reach many tens of thousands. For example, we estimated that there are ∼64 000 A1 Twitter neurons, based on the reported volume (8.18 mm^3^) and neuron density (78 080/mm^3^) of the marmoset A1 [12].

### Response properties of neuron populations for compound calls

**Figure 3.**
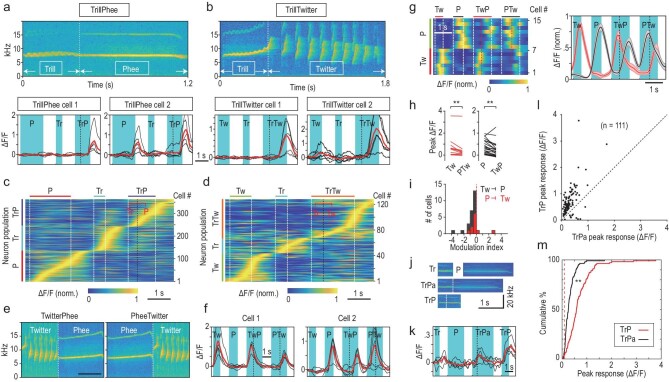
Properties of compound-call-selective cells. (a and b) Spectrograms of TrillPhee and TrillTwitter, and selective responses of two example cells for each compound call. (c and d) Heat maps of the activity of all cells selectively responding to compound calls ((c) M_a_, M_c_, M_d_, Cal-520AM-labeled; (d) TrillTwitter, M_a_, GCaMP6f-labeled) and simple calls (Trill, Phee, Twitter). Black dashed line, boundary of simple-call components. (e) Spectrograms of novel compound calls TwitterPhee and PheeTwitter. Bar: 1 s. (f) Single trials (black lines, n = 5) and mean (red line) evoked by Twitter, Phee, TwitterPhee and PheeTwitter in two example cells. Dashed line, boundary of Phee and Twitter. (g) Heat map of normalized responses to simple calls and artificial compound calls (left) for example cells that show selective response to Twitter (n = 7) and Phee (n = 8), and their responses to artificial compound calls. Right: normalized mean ΔF/F (± SEM) induced by simple calls and artificial compound calls for all cells of the Twitter (red) and Phee (black) neuron populations, corresponding to the heat map on the left. Note that both Twitter and Phee cells responded to artificial TwitterPhee and PheeTwitter with reduced amplitudes. (h) Comparison of the peak ΔF/F values for individual neurons within the Twitter (n = 11) and Phee (n = 26) neuron population, between responses to isolated simple calls and those to the same simple calls within artificial compound calls (**, *P* < 0.01, paired *t*-test). (i) The inhibitory effect of one simple call on another that followed immediately, as quantified by the modulation index (MI) that represents fractional changes in the peak ΔF/F of simple-call-evoked responses (see Methods). Note that MIs were predominantly negative for both Twitter and Phee neurons. (j) Spectrograms of natural Trill, Phee and artificial TrillPhee (TrPa) made from natural Trill and Phee, and a natural TrillPhee. All calls are from the same marmoset M_2_. (k) Single trials (black lines, n = 5) and mean (red line) evoked by Trill, Phee, TrPa and natural TrillPhee in an example cell. Dashed line: boundary of Trill and Phee. (l) Responses to natural TrillPhee and TrPa of 111 neurons. (m) Cumulative percentages of neurons that responded to natural and artificial TrillPhee with different amplitudes. Red dashed line, value of 0.1 in ΔF/F. The difference between two distributions is significant at *P* < 0.001, Kolmogorov-Smirnov test. P = Phee; Tw = Twitter; Tr = Trill; TrP = TrillPhee; TwP = TwitterPhee; PTw = PheeTwitter.

Further measurements of neuronal responses to two compound calls, TrillPhee and TrillTwitter, showed that response onset generally occurred after the appearance of the second simple call-like component (Fig. 3a and b, cell 1), and only a few of these compound-call neurons responded weakly to an isolated Phee or Twitter (Fig. 3a and b, cell 2). Activity heat maps of all TrillPhee neurons (Fig. 3c, M_a_, M_c_, M_d_; Cal-520AM-labeled) and TrillTwitter neurons (Fig. 3d, M_a_; GCaMP6f-labeled), as well as Trill, Phee and Twitter neurons, showed that the size of the compound-call population could be as large as a simple-call population.

We have also constructed artificial compound calls by linking two natural simple calls, Twitter and Phee, from the same marmoset M_0_ (Fig. 3e). We found that the novel compound calls TwitterPhee and PheeTwitter, which were never recorded in our marmoset colony, failed to elicit any compound-call-selective response. All neuronal responses appeared to be evoked by the simple call Twitter or Phee (Fig. 3f, M_a_ and M_d_), and the peak amplitudes of these artificial compound-call-evoked responses were slightly lower than those evoked by isolated Phee or Twitter (Fig. 3g–i), implicating the inhibitory action between simple calls. We also constructed artificial TrillPhee by joining two randomly sampled simple calls, Trill and Phee, recorded from M_2_, and found that such artificial TrillPhee could evoke significant selective responses in many A1 neurons, but the response amplitudes were consistently lower than those evoked by the natural TrillPhee made by the same animal, as shown by the example neuron (Fig. 3k) and all neurons examined (n = 111, Fig. 3l and m). This could be attributed in part to the difference in spectra-temporal profiles between the Trill- and Phee-like component within natural TrillPhee and those of isolated Trill and Phee calls.

Taken together, these results on artificial compound calls suggest that compound-call-selective neurons are developed in A1 for detecting natural compound calls, via natural selection or auditory experience, or both.

### Domain deletion, sequence alteration and interval extension of compound calls

**Figure 4.**
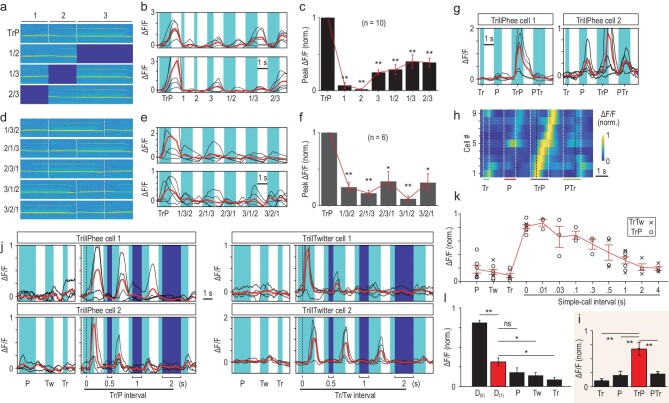
Experiments on ‘domain deletion’, ‘domain sequence alteration’, sequence reversal and interval extension of compound calls. (a) Spectrograms of a complete TrillPhee and domain-deleted TrillPhee, in which one of three domains (1, Trill; 2, Trill/Phee junction; 3, Phee) was deleted. (b) Two example TrillPhee neurons responding to the complete TrillPhee and one or two TrillPhee domains. (c) Summary of normalized peak ΔF/F values for all 10 TrillPhee cells examined in domain-deletion experiments. (d) Spectrograms of TrillPhee with domain sequence alteration, based on three domains defined in (a). (e) Two examples of TrillPhee cells responding to complete TrillPhee and five different domain sequence-altered TrillPhees. (f) Summary of normalized peak ΔF/F values for all six TrillPhee neurons examined in domain sequence alteration experiments. (g) Two examples of TrillPhee neurons showed complete loss of compound-call selectivity when the Trill/Phee sequence was changed to Phee/Trill. (h) Heat map of nine TrillPhee neurons examined in the ‘reverse sequence’ experiment, showing responses to TrillPhee but not PheeTrill. The ΔF/F value was normalized for each cell. (i) Summary of average peak ΔF/F values for all cells shown in (h) (**, *P* < 0.001, paired *t-*test). (j) Two example cells with selective responses to natural compound calls (left, TrillPhee; right, TrillTwitter) and reconstructed compound calls with an interval of 0.5, 1 or 2 s between two component simple calls, together with their responses to isolated simple calls Trill, Phee and Twitter. (k) Summary of all data on responses evoked by reconstructed compound calls with extended intervals from 0.01 to 4 s (n = 3–7 cells each) and by three isolated constituent simple calls, recorded from marmoset M_a_ expressing GCaMP6f. Red curve: averages at all intervals, with data points depicting the normalized peak value of ΔF/F for two compound calls. (l) Averages of normalized peak ΔF/F values for data in (k), for natural compound call (D_(0)_), extended compound call with 1-s interval (D_(1)_) and three constituent simple calls (n = 7 cells; paired *t*-test; **, *P* < 0.001; *, *P* < 0.01; ns, *P* > 0.05). P = Phee; Tw = Twitter; Tr = Trill; TrP = TrillPhee; PTr = PheeTrill.

Are call-selective responses of A1 neurons due to a unique spectral-temporal property of a specific sound domain within the call? We address this question by focusing on the compound call TrillPhee, which has a more complex spectrogram. In two GCaMP6f-expressing marmosets, we first performed ‘domain deletion’ experiments, in which three separate domains of the TrillPhee (D_1_: Trill; D_2_: Trill-Phee junction; D_3_: Phee) were sequentially deleted (Fig. 4a). We found that deleting either one or two domains within TrillPhee markedly reduced the response of TrillPhee neurons (Fig. 4b, two example cells; Fig. 4c, all 10 cells recorded in M_a_ and M_b_). This indicates that TrillPhee responses were due to a global rather than local spectral-temporal property of the call. In further ‘domain sequence alteration’ experiments, whereby the three TrillPhee domains were all present but their temporal sequence were altered in five different ways. We found that any alteration of the natural sequence (D_1_/D_2_/D_3_) resulted in marked reduction of evoked responses (Fig. 4e and f). Thus, both the presence of all domains and their proper temporal sequence are critical, implicating sequence-specific integration of information on different sound components by the call-responsive neurons. The importance of the temporal sequence of sound components was further confirmed by the finding that reversing the Trill/Phee sequence into Phee/Trill completely abolished the TrillPhee-selective responses in all nine of the TrillPhee neurons examined (Fig. 4g–i, marmoset M_a_).

In addition to domain sequence specificity, we further examined reconstructed compound calls in which the interval between simple call-like components was extended from 10 ms up to 4 s. The responses declined around an interval of ∼100 ms and largely disappeared beyond 1 s (examples, Fig. 4j; summary, Fig. 4k and l). Compound calls with over-extended intervals between simple call-like components still triggered weak responses in some compound-call neurons (for example, TrillPhee cell 2 in Fig. 4j). Thus, normal call-selective responses require not only the proper sequence of the simple call-like components, but also their temporal proximity within ∼1 s.

In a separate experiment, we monitored the activity of Phee-selective neurons with the imposition of a preceding Trill (isolated from a TrillPhee call) at intervals of 0, 0.5, 1 or 2 s, and found that the suppression effect of the preceding Trill gradually reduced as the interval of Trill/Phee was increased (Supplementary Fig. 9). Thus, simple call-induced suppression is also interval dependent.

### Effects of anesthesia on call-selective responses

**Figure 5.**
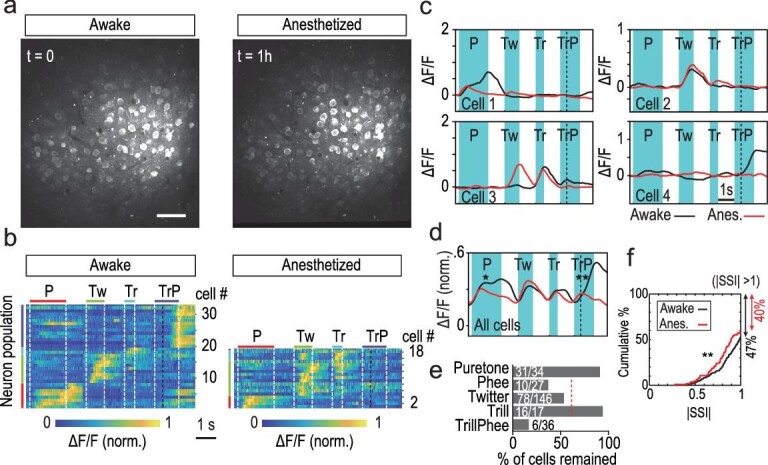
Anesthesia reduced call selectivity. (a) Images of Cal-520AM fluorescence (averaged over 2 min) at a recorded region in marmoset M_d_, before (left) and 1 h after (right) induction of light anesthesia with a fentanyl cocktail. (b) Heat maps of the activity of call-selective cells within an example imaging field (shown in (a)) in awake state and 1 h after anesthesia. Note that TrillPhee neurons largely disappeared after anesthesia (only two remained). (c) Four example cells depicting call-selective responses shown in (b) with each trace depicting averaged signals from five trials. (d) Summary of all data on call-selective cells (n = 62, three imaging fields, M_d_) before (black) and 1 h after (red) anesthesia, shown by the average traces of ΔF/F. The mean ΔF/F values after anesthesia were determined based on the normalization used for the same neuron in the awake state. Significant differences were found for TrillPhee and Phee neurons (Phee, *P* < 0.01; TrillPhee, *P* < 0.001; Twitter, Trill, *P* > 0.05; *t-*test). (e) The percentage of total cells that continued to show pure-tone, Phee, Twitter, Trill and TrillPhee responses, 1 h after anesthesia induction. Red dashed line, mean value of Phee, Twitter and Trill. Data were from M_b_ and M_d_. (f) Cumulative percentage plot of the distribution of absolute CSI values for all call-selective cells before (black line, n = 226, 47% of neurons showing CSI > 1) and 1 h after (red line, n = 112, 40% of neurons showing CSI > 1) anesthesia induction. Data were from M_b_ and M_d_. The difference between two distributions (including all cells) is significant at *P* < 0.05, Kolmogorov-Smirnov test. P = Phee; Tw = Twitter; Tr = Trill; TrP = TrillPhee.

Many previous studies of auditory processing in non-human primates were performed in anesthetized preparations [13–15]. In this study, we adopted a fentanyl cocktail for light anesthesia [16], under which pure-tone responses were still robustly evoked in A1 [6], and the overall level of Cal-520AM fluorescence remained largely unchanged (Fig. 5a). We found that this anesthesia modulated the responses of both simple-call and compound-call neurons. Many simple-call neurons still exhibited call-selective responses with lower amplitudes, but their temporal profiles were altered (Fig. 5b and c). Notably, a large proportion of TrillPhee neurons became completely non-responsive to TrillPhee (Fig. 5b and c, Supplementary Fig. 10). Comparison of response profiles of the same population of neurons before and during anesthesia showed anesthesia-induced reduction of amplitude, duration and call selectivity (Fig. 5d–f). Supplementary Fig. 10 shows the anesthesia responses for the same cell, for all cells recorded before and after anesthesia.

## DISCUSSION

Auditory processing in A1 is characterized by the tonotopic organization and spectral-temporal selectivity of neuronal responses [17,18], presumably involving feed-forward thalamocortical inputs and intracortical processing by local circuits [19–21]. Here we show that, in marmoset A1 tonotopic regions comprising neurons predominantly tuned to specific sound frequencies, there are substantial populations of neurons specifically devoted to call processing. To determine whether neurons are selective to natural calls, we first examined the responses of each A1 neuron to the same calls from different animals. We found that, despite some dispersion of spectra-temporal properties of the same call made by different marmosets, the same selective response pattern was evoked in the same neuron, indicating invariance of the responses to conspecific calls. Further studies using standard calls provided evidence for complex suppressive and facilitatory processing in call-evoked responses. First, we found that responses of simple-call-selective neurons were suppressed by the presence of other preceding simple calls, indicating suppressive interactions among neurons responding to simple calls. Second, the requirement of the specific sequence of call domains and the restricted interval between simple-call-like components within the compound call suggest well-orchestrated facilitatory modulation. Finally, the high susceptibility of compound-call-evoked responses to disruption by light anesthesia is consistent with the presence of polysynaptic signaling and top-down regulation, which are known to be more vulnerable to anesthesia [22,23].

An important issue of vocal communication is the processing of the temporal sequence and time interval of sound units that could span timescales from milliseconds to seconds [24]. Previous studies have shown that selectivity for conspecific sounds is present in the avian primary auditory forebrain, and spectral-temporal features of sounds could account for the neuronal responses. In marmoset A1, temporal compression, extension or reversion of marmoset Twitter calls greatly diminished neural firing evoked by the natural Twitter [10], indicating the importance of the temporal feature of the sound. Our findings are in line with these previous reports and further show that not only is the spectral-temporal structure of the sound over hundreds-of-millisecond timescales important, the sequence of sound components over a temporal window of seconds is also critical. Notably, call sound processing involves a continuous coding of sound information by responsive neurons. Our population recording data showed that such prolonged coding could be achieved by call-specific neuron populations with peak response profiles that tiled over a period of seconds, allowing continuous coding of the global spectra-temporal property as well as the sequence of sound components of the call.

A major finding of this study is the temporal context within which the call sound occurs, as shown by the suppressive and facilitatory actions among temporally conjunctive simple calls. For example, the immediate prior presence of Trill suppressed the Phee-evoked responses of Phee-selective neurons. Such suppression disappeared when the interval between Trill and Phee was extended beyond 1 s. Such suppression could be mediated by Trill neuron-activated interneurons that provide extended inhibitory inputs to Phee-selective neurons over a period of up to 1 s, covering the entire duration of the Phee sound via temporal tiling of Trill neuron responses. On the other hand, we found that in TrillPhee neurons, the immediate prior presence of a Trill-like sound appeared to facilitate the neuron's response to the subsequent Phee-like component. This could be accomplished by the Trill neuron activation that causes disynaptic disinhibition of Phee-evoked responses, if TrillPhee neurons are normally under strong inhibition that prevents their response to a Phee-like sound in the absence of a preceding Trill-like sound. It remains to be further determined whether these actions involve intracortical circuits within A1 or other regions of the auditory pathway, or both. A study using functional magnetic resonance imaging with macaques has shown preferential activity in anterior auditory fields for species-specific vocalization and vocal identification of conspecific macaque monkeys [25]. Neuronal representation of simple and compound calls in A1 could serve as building blocks for further circuit computation of more selective representation in higher cortical regions.

Vocal communication has been extensively studied using songbirds [26–28], rodents [29,30] and non-human primates [2,31]. In birds, neurons in the tonotopically organized primary auditory field and the caudal hyperstriatum ventral region (cHV) show extremely selective responses to the bird's own song but not conspecific songs by others [32]. In mice, neurons in the inferior colliculus and some auditory cortical regions respond robustly to ultrasonic vocalization [29]. Studies in humans and non-human primates have shown that neurons sensitive to conspecific vocal sounds exist in many regions of the superior temporal cortex [31], including A1. Thus, call-selective responses in A1 could reflect activity of down or upstream regions of the auditory pathway. Alternatively, these A1 call-selective neurons could be the main site of information processing underlying call recognition. Further experiments that examine the effect of silencing activity in different brain regions on A1 call-selective responses are required to explore these two possibilities. Our characterization of distinct types of call-selective A1 neurons at the population level offers a basis for analyzing the circuit processing of marmoset vocal sounds.

Neural circuit analysis of complex vocal sounds, including calls, phrases and sentences, is beginning to be addressed by advanced technologies that allow recording of population neuronal activity with high spatiotemporal resolution [33–37]. Simultaneous recording of spiking activity in multiple brain regions could further elucidate the spatiotemporal sequence of vocal sound signal processing in unanesthetized animals. In particular, long-term optical recording over large populations of neurons, together with optogenetic manipulation of circuit activity, could help to unravel circuit mechanisms underlying vocal sound processing and experience-dependent circuit plasticity. Developmental and social interaction-dependent changes of marmoset vocal sound production have been observed [38,39]. Whether vocal sound recognition also exhibits plasticity remains unclear. We found no selective response in A1 neurons towards unnatural compound calls when the marmoset was exposed to the latter over periods of minutes. It is possible that prolonged exposure under appropriate contexts could result in circuit modification that allows marmoset recognition of novel sounds, as suggested by the finding in mice that a sparse set of A1 neurons could become responsive to learned complex sounds [40].

## METHODS

Detailed materials and methods are available in the Supplementary Data.

## Supplementary Material

nwab126_Supplemental_Files

## References

[1] Miller CT , Freiwald WA, Leopold DA et al. Marmosets: a neuroscientific model of human social behavior. Neuron 2016; 90: 219–33. 10.1016/j.neuron.2016.03.018 27100195 PMC4840471

[2] Eliades SJ , Miller CT. Marmoset vocal communication: behavior and neurobiology. Devel Neurobio 2017; 77: 286–99. 10.1002/dneu.22464 27739195

[3] Sadagopan S , Temiz-Karayol NZ, Voss HU. High-field functional magnetic resonance imaging of vocalization processing in marmosets. Sci Rep 2015; 5: 10950. 10.1038/srep10950 26091254 PMC4473644

[4] Belin P , Bodin C, Aglieri V. A ‘voice patch’ system in the primate brain for processing vocal information? Hear Res 2018; 366: 65–74. 10.1016/j.heares.2018.04.010 29776691

[5] Scott SK , Johnsrude IS. The neuroanatomical and functional organization of speech perception. Trends Neurosci 2003; 26: 100–7. 10.1016/S0166-2236(02)00037-1 12536133

[6] Zeng HH , Huang JF, Chen M et al. Local homogeneity of tonotopic organization in the primary auditory cortex of marmosets. Proc Natl Acad Sci USA 2019; 116: 3239–44. 10.1073/pnas.1816653116 30718428 PMC6386663

[7] Tani T , Abe H, Hayami T et al. Sound frequency representation in the auditory cortex of the common marmoset visualized using optical intrinsic signal imaging. eNeuro 2018; doi: 10.1523/eneuro.0078-18.2018. 10.1523/ENEURO.0078-18.2018 PMC5937112 29736410

[8] Feng L , Wang X. Harmonic template neurons in primate auditory cortex underlying complex sound processing. Proc Natl Acad Sci USA 2017; 114: E840–8. 10.1073/pnas.1607519114 28096341 PMC5293092

[9] Yin P , Johnson JS, O’Connor KN et al. Coding of amplitude modulation in primary auditory cortex. J Neurophysiol 2011; 105: 582–600. 10.1152/jn.00621.2010 21148093 PMC3059165

[10] Wang X , Merzenich MM, Beitel R et al. Representation of a species-specific vocalization in the primary auditory cortex of the common marmoset: temporal and spectral characteristics. J Neurophysiol 1995; 74: 2685–706. 10.1152/jn.1995.74.6.2685 8747224

[11] Sadakane O , Masamizu Y, Watakabe A et al. Long-term two-photon calcium imaging of neuronal populations with subcellular resolution in adult non-human primates. Cell Rep 2015; 13: 1989–99. 10.1016/j.celrep.2015.10.050 26655910

[12] Atapour N , Majka P, Wolkowicz IH et al. Neuronal distribution across the cerebral cortex of the marmoset monkey (Callithrix jacchus). Cereb Cortex 2019; 29: 3836–63. 10.1093/cercor/bhy263 30357325

[13] Wang X. On cortical coding of vocal communication sounds in primates. Proc Natl Acad Sci USA 2000; 97: 11843–9. 10.1073/pnas.97.22.11843 11050218 PMC34358

[14] Wang X. Cortical coding of auditory features. Annu Rev Neurosci 2018; 41: 527–52. 10.1146/annurev-neuro-072116-031302 29986161

[15] Hubel DH , Henson CO, Rupert A et al. Attention units in the auditory cortex. Science 1959; 129: 1279–80. 10.1126/science.129.3358.1279 13658956

[16] Jaepel J , Hubener M, Bonhoeffer T et al. Lateral geniculate neurons projecting to primary visual cortex show ocular dominance plasticity in adult mice. Nat Neurosci 2017; 20: 1708–14. 10.1038/s41593-017-0021-0 29184207

[17] Bendor D , Wang X. The neuronal representation of pitch in primate auditory cortex. Nature 2005; 436: 1161–5. 10.1038/nature03867 16121182 PMC1780171

[18] Bendor D , Wang X. Cortical representations of pitch in monkeys and humans. Curr Opin Neurobiol 2006; 16: 391–9. 10.1016/j.conb.2006.07.001 16842992 PMC4325365

[19] Zhang LI , Tan AY, Schreiner CE et al. Topography and synaptic shaping of direction selectivity in primary auditory cortex. Nature 2003; 424: 201–5. 10.1038/nature01796 12853959

[20] Li LY , Li YT, Zhou M et al. Intracortical multiplication of thalamocortical signals in mouse auditory cortex. Nat Neurosci 2013; 16: 1179–81. 10.1038/nn.3493 23933752 PMC3844430

[21] Hamilton LS , Sohl-Dickstein J, Huth AG et al. Optogenetic activation of an inhibitory network enhances feedforward functional connectivity in auditory cortex. Neuron 2013; 80: 1066–76. 10.1016/j.neuron.2013.08.017 24267655 PMC3841078

[22] Gilbert CD , Sigman M. Brain states: top-down influences in sensory processing. Neuron 2007; 54: 677–96. 10.1016/j.neuron.2007.05.019 17553419

[23] Nourski KV , Steinschneider M, Rhone AE et al. Auditory predictive coding across awareness states under anesthesia: an intracranial electrophysiology study. J Neurosci 2018; 38: 8441–52. 10.1523/JNEUROSCI.0967-18.2018 30126970 PMC6158689

[24] Mauk MD , Buonomano DV. The neural basis of temporal processing. Annu Rev Neurosci 2004; 27: 307–40. 10.1146/annurev.neuro.27.070203.144247 15217335

[25] Petkov CI , Kayser C, Steudel T et al. A voice region in the monkey brain. Nat Neurosci 2008; 11: 367–74. 10.1038/nn2043 18264095

[26] Theunissen FE , Shaevitz SS. Auditory processing of vocal sounds in birds. Curr Opin Neurobiol 2006; 16: 400–7. 10.1016/j.conb.2006.07.003 16842993

[27] Prather JF , Mooney R. Neural correlates of learned song in the avian forebrain: simultaneous representation of self and others. Curr Opin Neurobiol 2004; 14: 496–502. 10.1016/j.conb.2004.06.004 15321071

[28] Schiavo JK , Valtcheva S, Bair-Marshall CJ et al. Innate and plastic mechanisms for maternal behaviour in auditory cortex. Nature 2020; 587: 426–31. 10.1038/s41586-020-2807-6 33029014 PMC7677212

[29] Egnor SR , Seagraves KM. The contribution of ultrasonic vocalizations to mouse courtship. Curr Opin Neurobiol 2016; 38: 1–5. 10.1038/s41586-020-2807-6 10.1038/s41586-020-2807-6 26789140

[30] Banerjee A , Phelps SM, Long MA. Singing mice. Curr Biol 2019; 29: R190–1. 10.1016/j.cub.2018.11.048 30889384

[31] Ghazanfar AA , Eliades SJ. The neurobiology of primate vocal communication. Curr Opin Neurobiol 2014; 28: 128–35. 10.1016/j.conb.2014.06.015 25062473 PMC4177356

[32] Grace JA , Amin N, Singh NC et al. Selectivity for conspecific song in the zebra finch auditory forebrain. J Neurophysiol 2003; 89: 472–87. 10.1152/jn.00088.2002 12522195

[33] Chang EF. Towards large-scale, human-based, mesoscopic neurotechnologies. Neuron 2015; 86: 68–78. 10.1016/j.neuron.2015.03.037 25856487 PMC5810414

[34] Yi HG , Leonard MK, Chang EF. The encoding of speech sounds in the superior temporal gyrus. Neuron 2019; 102: 1096–110. 10.1016/j.neuron.2019.04.023 31220442 PMC6602075

[35] Lu J , Li C, Singh-Alvarado J et al. MIN1PIPE: a miniscope 1-photon-based calcium imaging signal extraction pipeline. Cell Rep 2018; 23: 3673–84. 10.1016/j.celrep.2018.05.062 29925007 PMC6084484

[36] Katlowitz KA , Picardo MA, Long MA. Stable sequential activity underlying the maintenance of a precisely executed skilled behavior. Neuron 2018; 98: 1133–40. 10.1016/j.neuron.2018.05.017 29861283 PMC6094941

[37] Peh WY , Roberts TF, Mooney R. Imaging auditory representations of song and syllables in populations of sensorimotor neurons essential to vocal communication. J Neurosci 2015; 35: 5589–605. 10.1523/JNEUROSCI.2308-14.2015 25855175 PMC4388922

[38] Gultekin YB , Hage SR. Limiting parental feedback disrupts vocal development in marmoset monkeys. Nat Commun 2017; 8: 14046. 10.1038/ncomms14046 28090084 PMC5241798

[39] Gultekin YB , Hage SR. Limiting parental interaction during vocal development affects acoustic call structure in marmoset monkeys. Sci Adv 2018; 4: eaar4012. 10.1126/sciadv.aar4012 29651461 PMC5895450

[40] Wang M , Liao X, Li R et al. Single-neuron representation of learned complex sounds in the auditory cortex. Nat Commun 2020; 11: 4361. 10.1038/s41467-020-18142-z 32868773 PMC7459331

