# Supplementary material for: Distinct neuron populations for simple and compound calls in the primary auditory cortex of awake marmosets
Source: Natl Sci Rev. 2021 Jul 12;8(11):nwab126. doi: 10.1093/nsr/nwab126 (PMC8645005; doi:10.1093/nsr/nwab126)
Supplement: nwab126_Supplemental_Files [file nwab126_supplemental_files.zip › Supplementary data revision0608.docx]

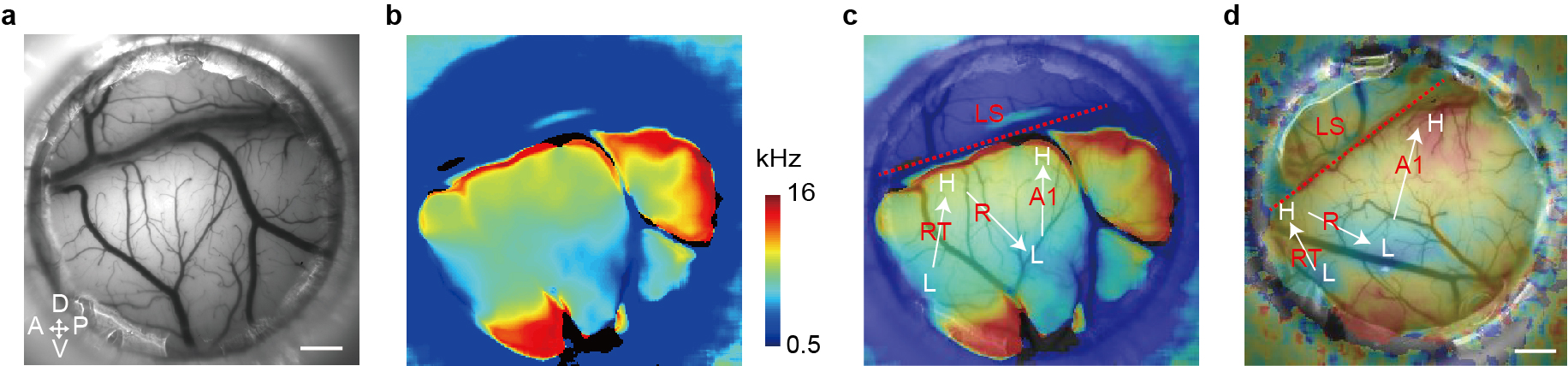


**Supplementary Figure 1 | Tonotopic map of the auditory cortex obtained by imaging intrinsic optical signals.**

**a**, Blood vessel map within the imaging window (M_a_). **b**, A tonotopic map revealed by intrinsic optical signals in response to a sequence of 21 discrete pure tone stimuli in the range of 0.5-16 kHz. The frequency preference of different imaged regions was color-coded by the scale bar. **c**, Image obtained by merging images in **a** and **b**. **d**, Same as **c** for another marmoset (M_d_). LS, lateral sulcus; A1, primary auditory cortex; R, rostral field; RT, rostro-temporal field. Bars: 1 mm.


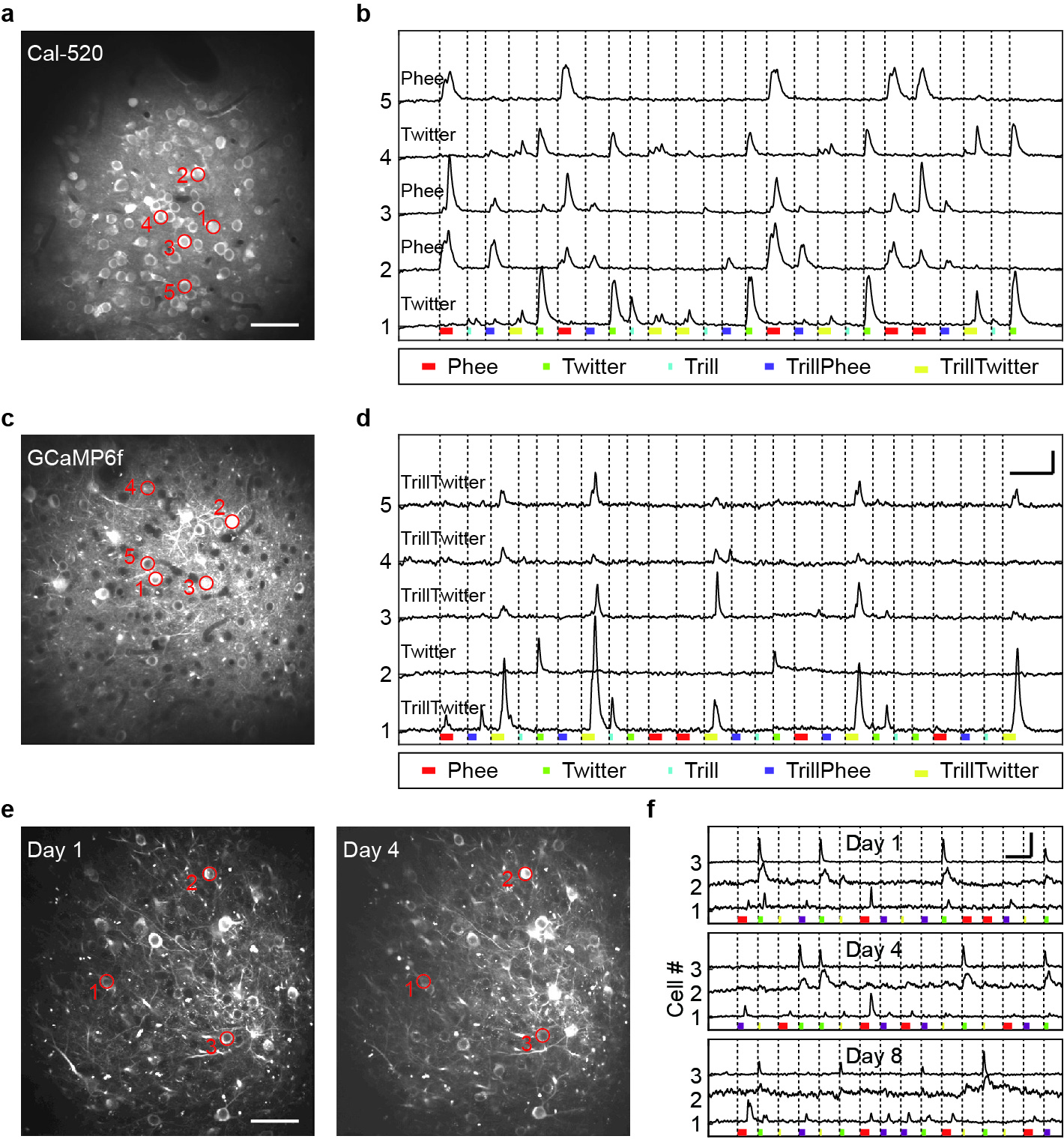


**Supplementary Figure 2 |** **Two-photon imaging of neuronal activity in awake marmoset A1.**

**a**, Fluorescence image of an A1 area of marmoset M_a_, loaded with Cal-520AM. Bar: 50 μm. **b**, Relative changes in fluorescence (ΔF/F) in 5 example cells (marked by circles in **a**) in response to 5 different calls in a random sequence. Stimulus duration marked by the bar below, call types coded in colors. The apparent call selectivity was assigned to each cell (marked on the trace). **c**, **d,** Similar to **a** and **b**, except that the A1 area was injected with Tet-dependent AAV expressing GCaMP6f. Data from marmoset M_a_. Bars: 5 s and 100% ΔF/F. **e**, Fluorescence image of an A1 area of a GCaMP6f-expressing marmoset (M_d_) on day 1 and day 4 of the experiment, which began 35 days after AAV injection, and 3 days after Tet application. Bar: 50 μm. **f**, Response profiles of 3 example neurons, marked by red circles in **e** and recorded at day 1, 4 and 8, showing the same apparent call selectivity. Bars: 5 s and normalized ΔF/F.


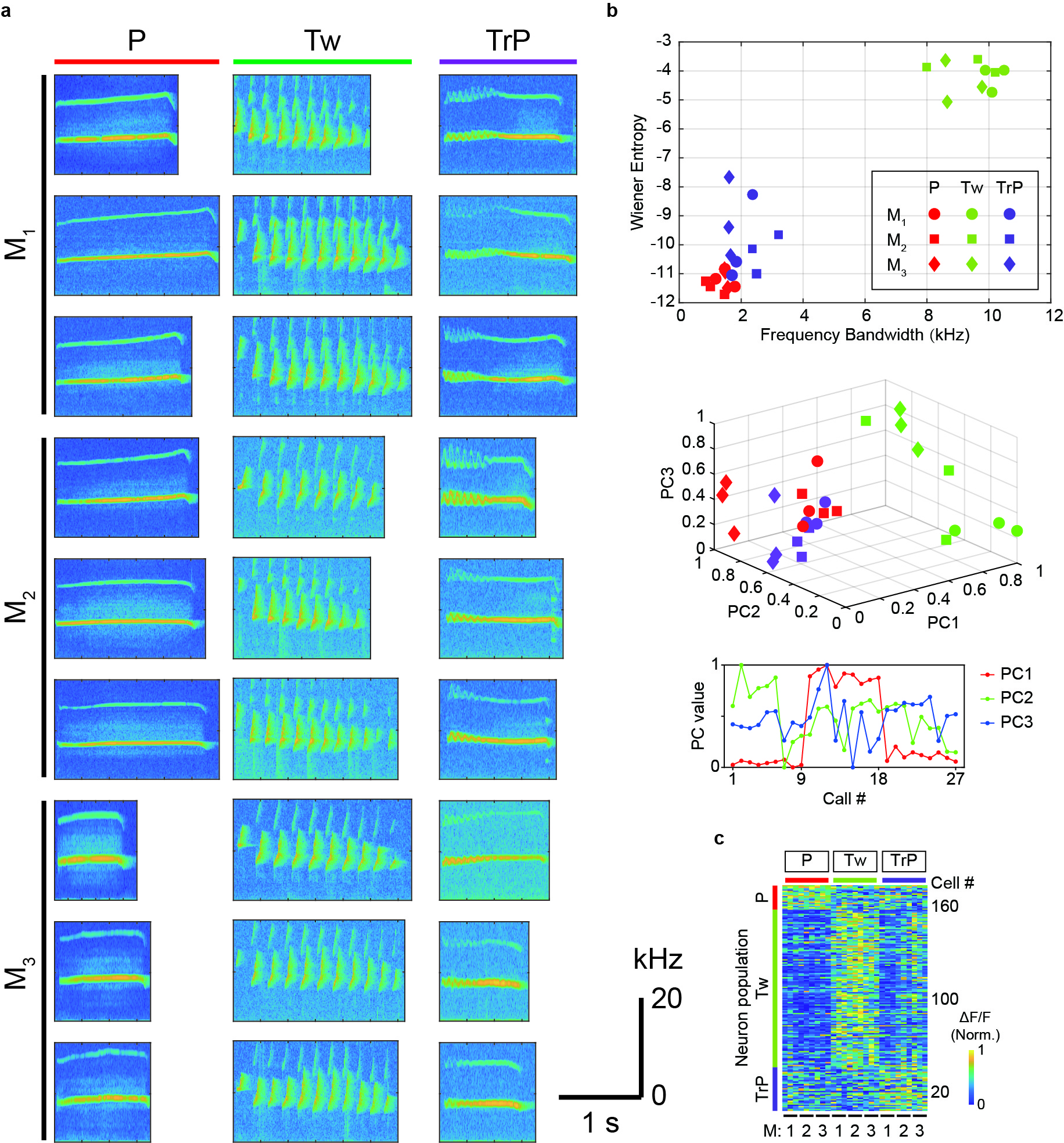


**Supplementary Figure 3 | Spectrograms of all 27 test calls from 3 marmosets (M_1_, M_2_, M_3_).**

**a**, Spectrograms of 27 call stimuli. **b**, Bandwidth Wiener-entropy analysis and principal component analysis (PCA) of 27 call stimuli shown in **a.** Shown in the middle panel and bottom are the first three principal components (PCs). **c**, Heat map for all call responsive neurons in marmoset M_b_ that was exposed to three call categories as in **a**. Each horizontal line depicts average amplitude of ΔF/F (from 5 trials), with 3 representative calls from each marmoset for each call. The cells were sorted into three call-selective neuron populations, based on the call category that evoked the maximal mean ΔF/F amplitude. The amplitude is coded in color by the scale shown on the right.

**
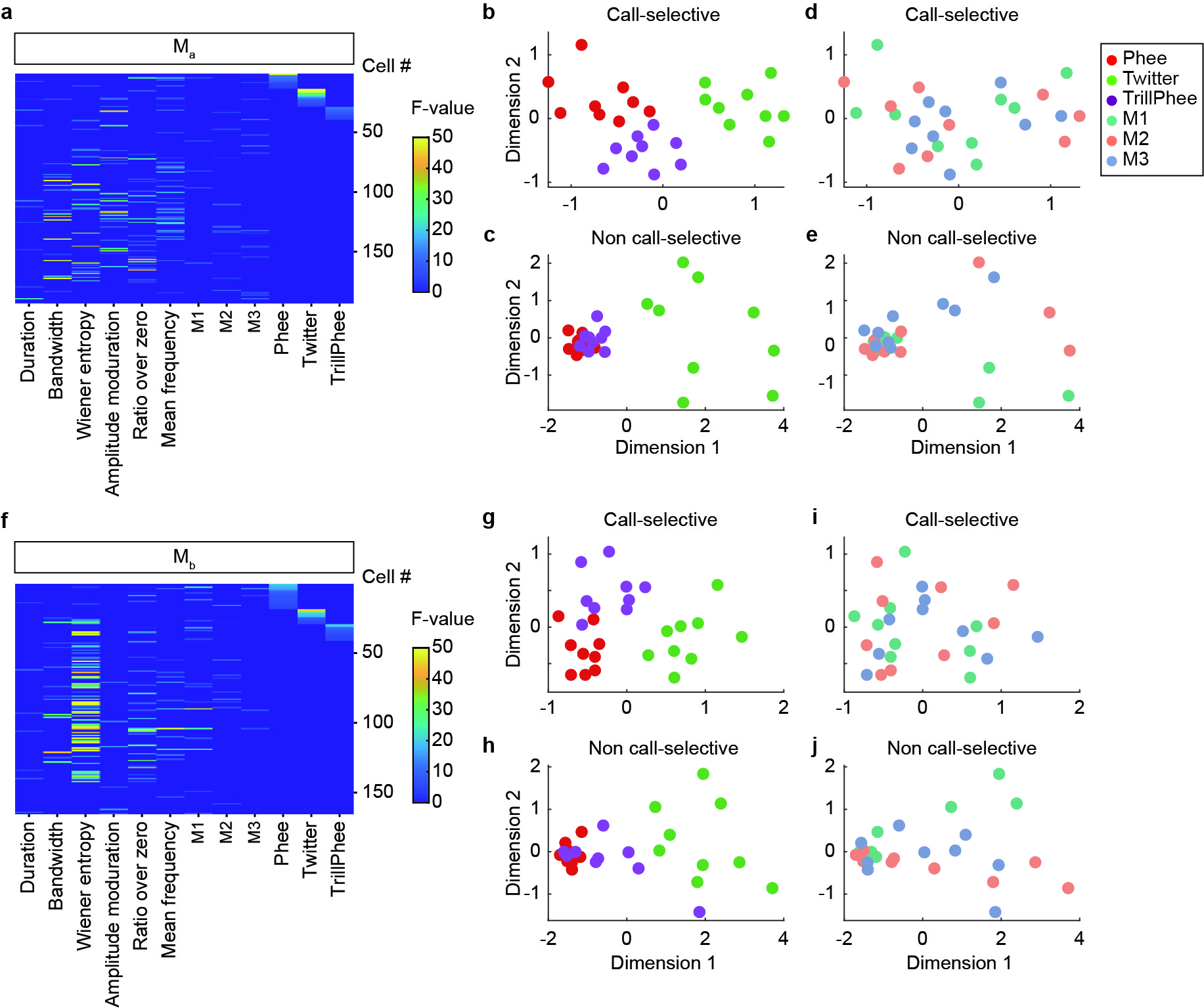
**

**Supplementary Figure 4 | a, Quantification of the relative selectivity of call-selective cells to specific acoustic features, animal identity, and call variables in the stimulus, using a generalized linear model (see Methods)**. For each variable, the column depicts the significance level for response modulation by that variable of all neurons from M_a_ recorded in experiments shown in Fig. 1. The neurons were sorted according to their call selectivity. **b**, Multi-dimensional scaling plot of neuronal representation of the 27 calls (shown only for two dimensions) by the subsets of the neurons depicted in a, based on their selectivity: neurons that responded exclusively to calls and invariant to other variables (n = 25/193); call-selective neurons that also responded to non-call variables (**c**, n = 95/193). **d** & **e**, Same neural representations using the label of animal caller disrupted such neural clustering. Each dot denotes one call. **f**-**j**, Same depiction as **a**-**e**, except that the data were from M_b_ (n = 21/165, g; n = 85/165, h). Note distinct clusters for three calls in neurons that responded exclusively to call variable in both marmosets.


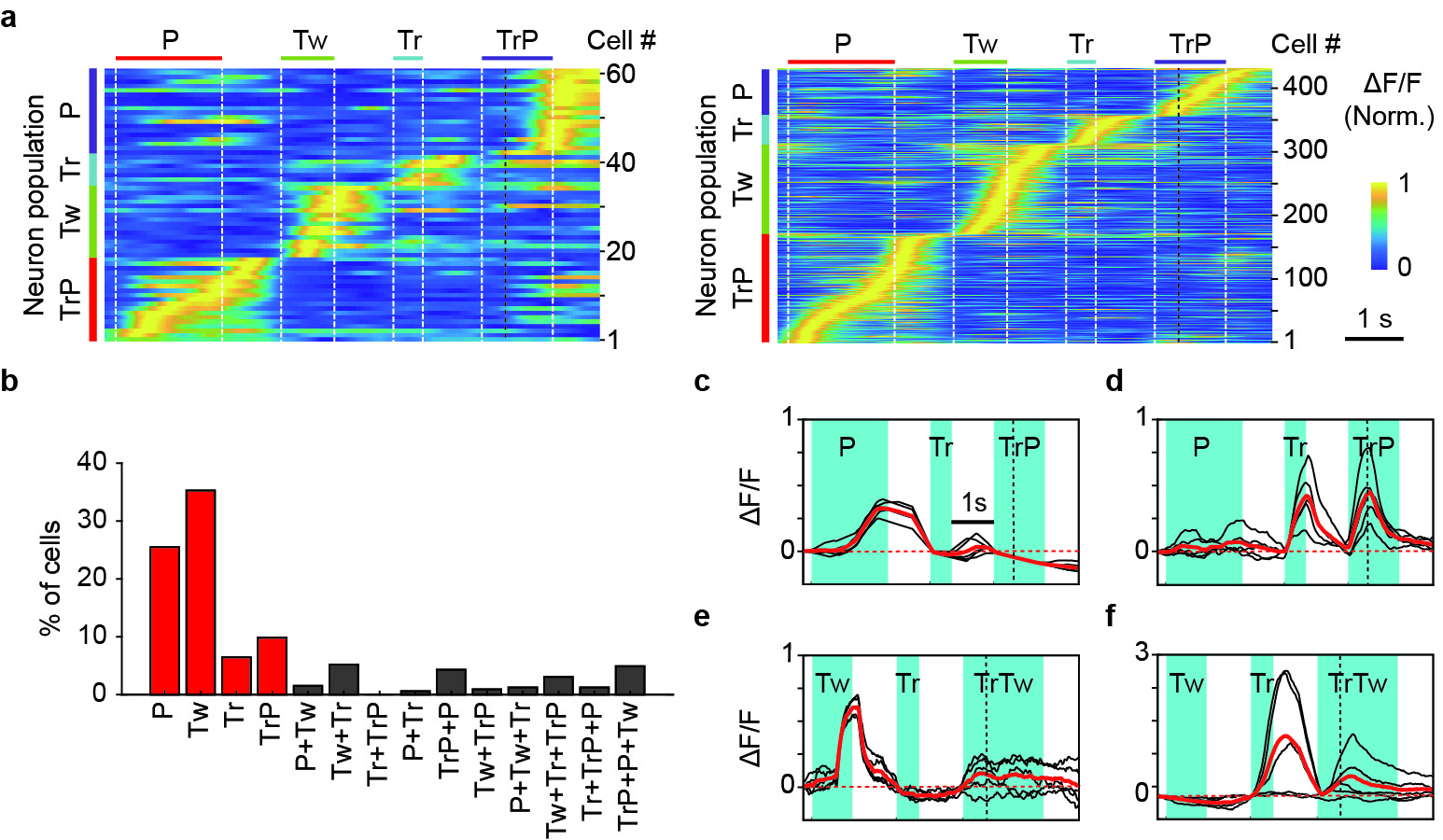


**Supplementary Figure 5 |** **Summary of call-selective neuron populations from two other marmosets recorded (M_a_, M_d_).**

**a**, Activities of all call-selective neurons recorded from marmoset M_d_ (left) and M_a_ (right) labeled with Cal-520AM, shown by the heat map in the same manner as in Fig. 1c. **b**, Percentages of cells showing selectivity to single call and to different sets of multiple calls, among all call-selective cells. **c** and **d**, Fluorescence changes (ΔF/F; black, individual trials; red, average) evoked by TrillPhee (TrP) and its component simple call Phee (P) and Trill (Tr), in two neurons recorded from marmoset M_c_. Red dash lines indicate zero ΔF/F. Note that this cell (**c**) exhibits negative ΔF/F to TrillPhee. **e** and **f**, Similar to **c** and **d**, but for responses evoked by TrillTwitter (TrTw), Trill (Tr) and Twitter (Tw). Neurons recorded from marmoset M_c_.


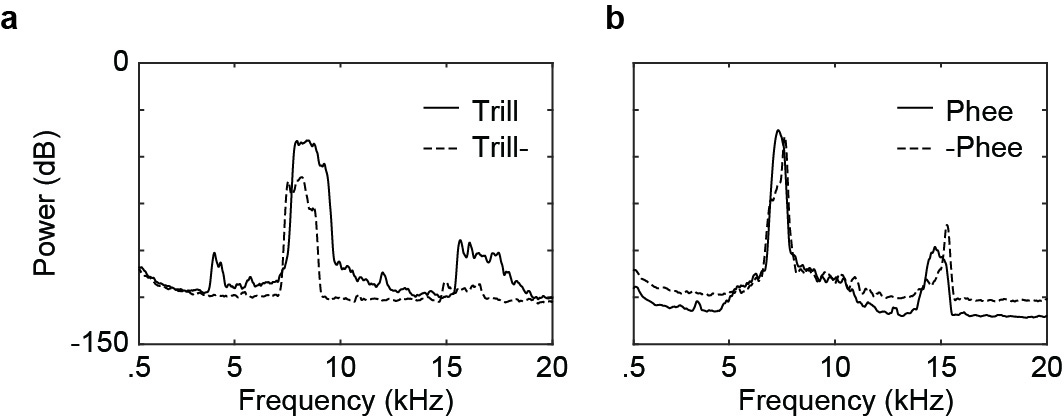


**Supplementary Figure 6 |** **Trill and Phee components in TrillPhee have similar dominant frequency but narrower frequency bandwidth compared to natural Trill and Phee.**

**a**, Power spectrum of natural Trill and Trill component (Trill-) in natural TrillPhee. **b**, Power spectrum of natural Phee and Phee component (Phee-) in natural TrillPhee.


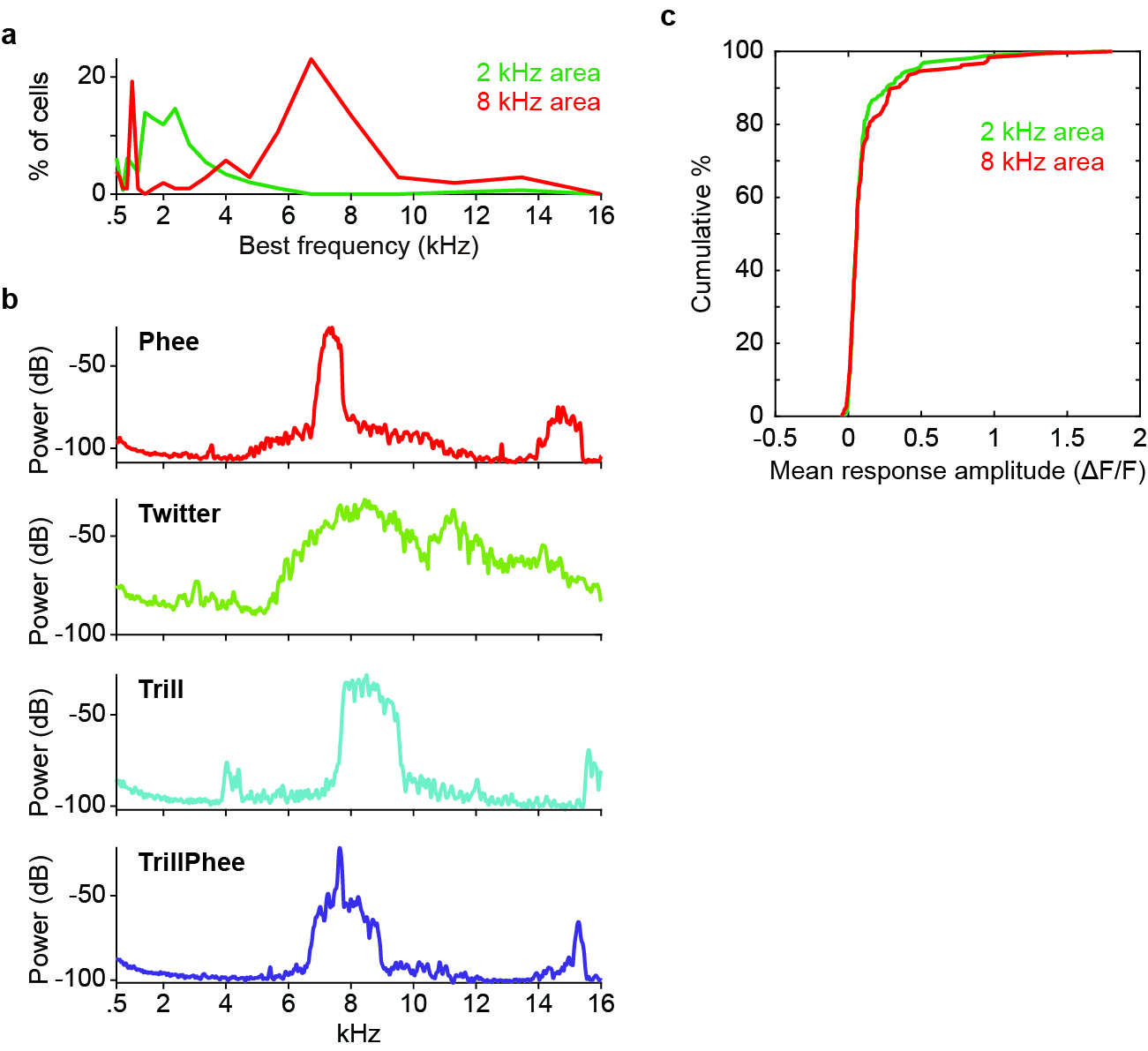


**Supplementary Figure 7 |** **Call-selective responses in 8 kHz area are slight larger than in 2 kHz area.**

**a,** The percentage of pure-tone responsive neurons showing different best frequencies. **b**, Power spectrum of 4 standard calls (Phee, Twitter, Trill and TrillPhee). Note all 4 calls have dominant frequency around 8 kHz. **c**, Cumulative percentages of neurons that selectively responded to 4 standard calls with different amplitudes. The difference between two distributions is nonsignificant (*P* = 0.857, *Kolmogorov-Smirnov* test).

**
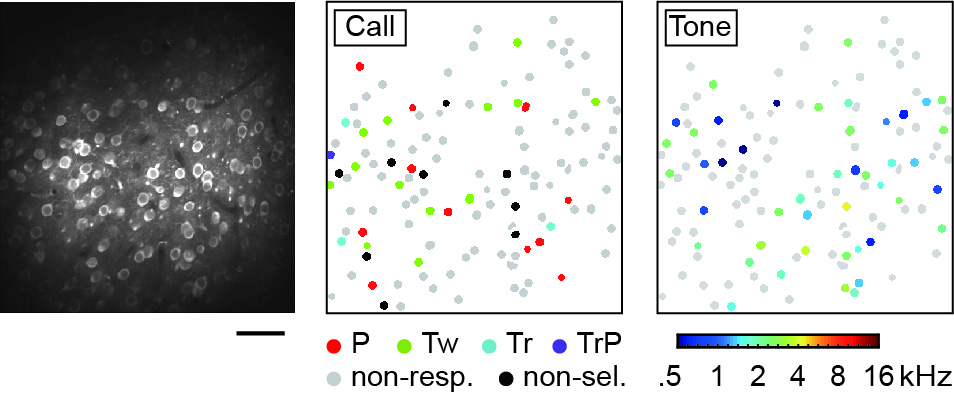
**

**Supplementary Figure 8 |** **Responses to calls and pure tones of A1 neuron populations in 2 kHz area.**

Left, an image of Cal-520AM fluorescence at a recorded region of marmoset M_c_. Bar: 50 μm. Middle, spatial distribution of all call-selective cells in the imaging field, with response properties (“non-resp.”, not responding to calls; “non-sel.”, non-selective responses to calls) coded in colors. Right, tone-selective cells within the same imaging field, with tone frequency coded in color scale shown below.

**
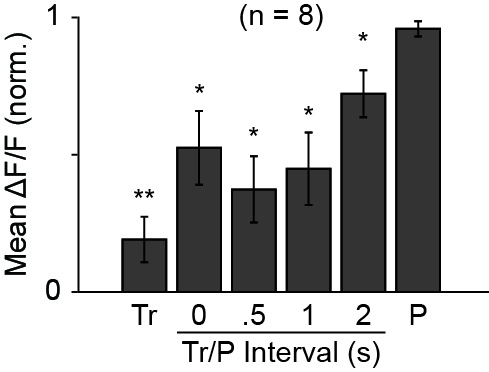
**

**Supplementary Figure 9 |** Suppression of Phee-evoked responses of Phee-selective neurons by a preceding Trill isolated from a natural TrillPhee call. The data represent mean peak ΔF/F values evoked in Phee-selective neurons, observed when the Trill/Phee interval was set at 0, 0.5, 1.0 and 2.0 s, together with responses evoked by Trill alone in these Phee-selective neurons (error bar, SEM; n = 8 cells). The mean amplitudes for Trill- or Trill/Phee-evoked responses were significantly smaller than those evoked by Phee alone (paired *t* test; **, *P* < 0.001; *, *P* < 0.05).


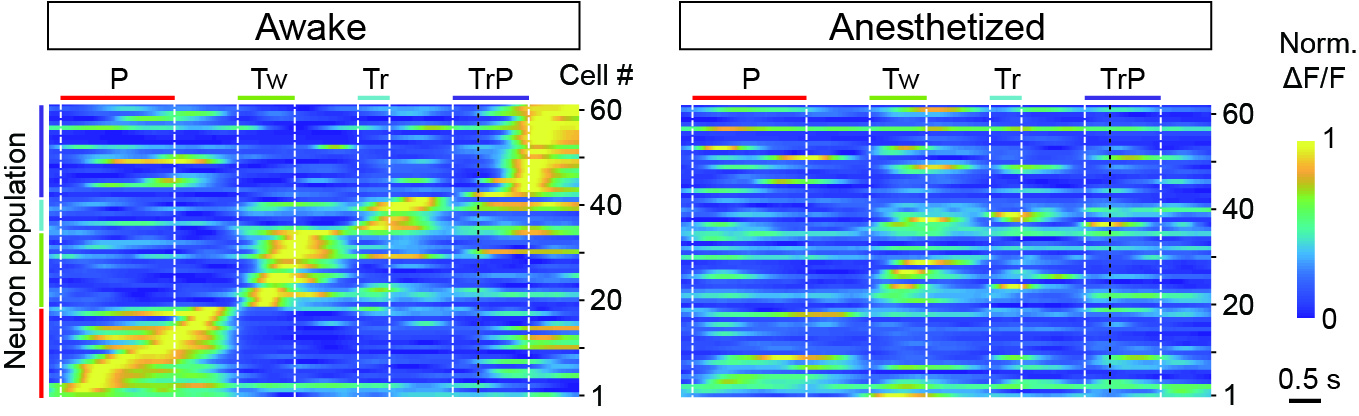


**Supplementary Figure 10** | Side by side comparing of response profiles of call-selective neurons recorded in awake and anesthesia, showing the anesthesia responses for the same neurons and sorted the same way as in the awake.
