# Supplementary material for: Distinct neuron populations for simple and compound calls in the primary auditory cortex of awake marmosets
Source: Natl Sci Rev. 2021 Jul 12;8(11):nwab126. doi: 10.1093/nsr/nwab126 (PMC8645005; doi:10.1093/nsr/nwab126)
Supplement: nwab126_Supplemental_Files [file nwab126_supplemental_files.zip › SupplementaryData_Methods_Final0608.docx]

**Methods**

**Animals.** Animal care and experimental procedures were approved by the Animal Care Committee of Shanghai Institutes for Biological Sciences, Chinese Academy of Sciences. Four adult common marmosets (*Callithrix jacchus*; Marmoset M_a_, M_b_, M_c_ and M_d_; 2 male and 2 females; body weight: 350-430g) provided by the non-human primate facility of the Institute of Neuroscience were used for imaging in this study. Six other marmosets (M_0_ - M_5_), living in different rooms from neural recording marmoset, were used for recording call samples. The marmosets were individually housed in a temperature- and humidity-controlled facility (26 - 30°C, 12 h light/dark cycles), and supplied with *ad libitum* water and balanced diet.

**Surgery.** Prior to the surgery, the marmoset was first injected with a fentanyl cocktail [1] intramuscularly (in mg/kg: 0.0005 fentanyl citrate, 0.5 midazolam and 0.05 dexmedetomidine). Afterwards, anesthesia was maintained by 1.5 - 3% isoflurane with pure oxygen, and the animal was kept in a customized frame in a prone position, with body temperature maintained at ~37°C (monitored with a rectal probe) using a heating blanket. During the surgery, the anesthesia state was confirmed by the absence of pinch-induced paw reflexes. A titanium head-post was attached to the parietal bone by dental cement, performed under sterile conditions. A circular craniotomy (window diameter 8 mm) and durotomy were performed to expose the auditory cortex. The custom-made chronic window consisted of a titanium ring, with coverslip (8 mm in diameter and 0.2 mm in thickness) glued by silicone adhesive (KN-300X, Kanglibang, China), and implanted to the scalp with dental cement seal. The animals were allowed to recover for at least 7 days after surgery. Antibiotics were intramuscularly administered for 3 consecutive days after surgery.

**Cal-520AM Loading.** For dye-loading experiments, marmosets were anesthetized by 1.5 - 2% isoflurane with pure oxygen through a custom-made mask. The chronic glass window was removed and replaced with a new glass window with a pinhole (~500 μm in diameter), which allowed the cortical access of a dye-loading glass micropipette. For preparing injection solution, Cal-520 AM (40-50 μg, AAT Bioquest) was dissolved with 4 μl Pluronic/DMSO mixture (20% w/v Pluronic F-127 in dimethyl sulfoxide) and was diluted with 35 μl of pipette solution (in mM: 10 HEPES, 2.5 KCl, 150 NaCl, pH 7.4.) and added with 1 μl of Alexa Fluor 594 solution (2 mM) for marking the injected solution. The injection solution was sonicated and filtered with a 0.22 μm filter to remove dye aggregates before loaded into the micropipette (2-4 μm tip opening), and pulse pressure-ejected (3-12 psi, 60-90 pulse at 1 Hz) with a Pico spritzer (Parker Hannifin, USA) to the layer 2/3 of A1 (200-300μm from the surface), as previously described [2]. The surgery and dye ejection usually were performed within 1 hr. After dye ejection, a new glass window without the pinhole was replaced and sealed. The animal was allowed to recover for at least 2 hr before 2-photon calcium imaging was performed when the animal was in the awake state, as indicated by readily licking the milk and eye tracking the experimenter’s action.

**Virus Injection.** Injection of GCaMP6f viral vector (for M_b_, M_c_ and M_d_) followed the procedure previously described [3]. In brief, after anesthesia, the chronic window was removed and a glass micropipette (30-μm tip opening) containing 3-4 μl of virus solution was inserted into A1 at a depth of 300-500 μm. The injected viral solution contained the Tetracycline (Tet)-on expression system [3] consisting of rAAV2/9-hSyn-rtTA (at final titer of 1-3×10^12^ vg/ml) and rAAV2/9-TRE3-GCaMP6f (final titer 2-8×10^12^ vg/ml) as equal-volume mixture. After virus injection (performed within 10 min), a new chronic glass window was installed. The two-photon imaging began ~4 weeks after injection, and GCaMP6f expression was induced by oral Tet administration (doxycycline 0.6 mg/ml in a 5% sucrose solution, 3-5 ml/d) for 3 d prior to the imaging experiment.

**Acoustic stimuli.** Marmoset calls were recorded from the marmoset colony at the non-human primate platform of Institute of Neuroscience, Chinese Academy of Sciences, from juvenile or adult marmosets individually in a soundproof chamber (at 48-kHz sampling frequency). Calls (Phee, Twitter and TrillPhee) used in call-invariant experiment (Fig. 1) were from marmoset M_1_, M_2_ and M_3_. For experiments described in Fig. 1, Trill was not tested in order to reduce the total experimental time of each session, the number of stimuli required to test was much larger (27 calls with 5 repeats each) than experiments described in other figures. Since Phee and Twitter calls are most common simple calls used in previous studies, we have thus chosen these two simple calls. Four standard test calls were sampled from marmoset M_0_ (Phee, Twitter and TrillPhee) and M_5_ (Trill). TrillTwitter was sampled from marmoset M_4_. Call stimuli were randomized and presented for 4-5 times with 1-4 s inter-stimulus intervals. For tonotopic mapping with intrinsic optical imaging, pure-tone stimuli (0.5-16 kHz, 4 clicks, 0.2 s duration and 0.5 s interval) were generated using MATLAB (MathWorks) without compression and exposed to the marmoset (48-kHz sampling frequency, 16 bits). The sound delivery system was calibrated using B&K calibrator (2669-L). The sound stimuli were presented by a KEF-ls50 speaker at a distance of 15 cm to the contralateral ear. Sinewave ramping (5-10 ms) was used in the onset and offset of sound stimuli. The sound intensities used in experiments were 70 dB SPL for both pure tones and calls. The customized sound-insulating chamber used here can attenuate most ambient noise (to a level < 30 dB SPL).

**Spectral-temporal analysis of calls.** To measure the spectral-temporal features of three calls (Phee, Twitter and TrillPhee) made by conspecific marmosets, we used a customized MATLAB script to analyze 27 calls from 3 marmosets (M_1_, M_2_, M_3_), with 3 calls for each call category by each marmoset. We first zero-phase bandpass (3 to 16 kHz) filtered the 27 calls and then measured the following features: duration, amplitude modulation (AM), frequency bandwidth, Wiener entropy, mean frequency and zero-crossing rate (ZCR). Amplitude modulation was calculated by applying Hilbert transform to call signals to obtain its envelope and further calculating the frequency of the envelope with maximum power. Frequency bandwidth represents the spectrum width of the entire call signal. The bandwidth threshold was set as 1% of the maximum power of the spectrum. Wiener entropy characterizes the width and uniformity of the spectrum and was calculated as the logarithm of the ratio between geometric and arithmetic means of the spectrum [4]. Mean frequency was calculated as the average of frequency from 500 to 24000Hz weighted by their relative power strength of the entire call. Since ZCRs were different across the call signal, we represented this feature by using the ratio of ZCRs between the first and last 100 ms of the call. Principal component analysis (PCA) was applied to reduce the number of dimensions in the feature, and the first three principal components (PCs) contributed to 98% of the variance.

**Imaging intrinsic optical signals.** Marmosets were anesthetized by 1.5 - 2.0% isoflurane with pure oxygen through a custom-made mask. The head was immobilized with a customized frame. During imaging sessions, the anesthesia was switched to intraperitoneally injection of a fentanyl cocktail (at mg/kg: 0.001 fentanyl citrate, 1 midazolam and 0.1 dexmedetomidine). Signals of reflectance change (intrinsic hemodynamic signals) corresponding to local cortical activity were acquired (Imager 3001, Optical Imaging Inc., Germantown, NY) with 660-nm illumination. Signal-to-noise ratio was enhanced by averaging data from many trials (15–30 trials per stimulus condition). Acoustic stimuli were presented in blocks, with each block containing all frequencies tested or no sound as control. For each condition, imaging began 0.6 s before the sound stimulus onset (for baseline signals). The total imaging time for each frequency was 6 s, during which 30 consecutive frames were collected (at 5 Hz). All stimulus frequencies were displayed in a randomized order.

**In vivo two-photon calcium imaging.** Marmosets were progressively trained for about two weeks for habituation in the head-fixed condition in an individually customized frame in the awake state. Fluorescent calcium signals, which are known to correlate with neuronal spiking activity [5, 6], were monitored from individual A1 neurons with a semi-custom-made LotosScan microscope (LotosScan, Suzhou Institute of Biomedical Engineering and Technology) coupled to a mode-locked Ti:Sa laser (Chameleon VISION-S, Coherent). The excitation wavelength was fixed at 920 nm. Imaging was performed using a 40X, 0.8 NA objective (Nikon). The beam size was large enough to cover the back aperture of the 40X objective. Images were acquired at a frame rate of 40 Hz. In experiment studying the effect of anesthesia, imaging was performed before and one hour after the induction of anesthesia, using the same anesthesia procedure described above.

**Data analysis.** Images were analyzed in MATLAB (MathWorks) and ImageJ (National Institutes of Health). For correcting the effect of lateral motion on the imaged data, a rigid-body transformation-based frame-by-frame alignment was applied by using Turboreg plugin (ImageJ software). Neurons were manually identified based on size and shape; astrocytes were identified and excluded based on their distinct morphology [7]. Fluorescence intensity changes with time for each neuron were extracted by averaging pixel intensity values within the cell mask (manually defined) in each frame. Neuropil signal was subtracted by using the method previously reported [8]. After this correction, fluorescence intensity change (ΔF) with time during the stimulus and the post-stimulus period (0.2 s for pure tones, 0.5 s for calls) were normalized by the pre-stimulus baseline fluorescence (F, over 0.2 s). For each stimulus, the mean ΔF/F was calculated by averaging ΔF/F over the entire stimulus duration for all trials of each stimulus condition. Cells showing significant differences in mean ΔF/F during the baseline vs. stimulus-presentation period (*p* < 0.05, ANOVA) were defined as “responsive cells”. Among them, call-selective cells were further defined by having responses to some calls that were significantly higher than those to other calls (*p* < 0.05, ANOVA) [9].

To further confirm the call-selective neurons were exclusively responsive to specific calls rather than statistically biased by the acoustic features compounding the calls, i.e. call frequency band or amplitude which co-varies with the calls, a step-wise GLM (Generalized Linear Model) was conducted. The feature matrix X included eleven predictors which were five acoustic features: duration (s), sound frequency bandwidth (kHz), Wiener entropy, amplitude modulation (Hz) and the ratio over zero from the call onset to offset; and six dummy variables (one-hot) representing the three monkeys’ identity, and the three calls respectively. For each neuron, its activity was taken as the response variable y and a stepwise GLM was run to find out which predictors more significantly contributed to the response variability: starting from a constant model, each time a predictor would be added to the model if it explained the most remaining deviance in the responses. A threshold p<0.05 was taken, and the F-values of the significant predictors per neuron were plotted in Figure 2i (non-significant cases were set to zero) and sorted according to the call-selectivity, in descending order. In total, Monkey M_a_ had 550 neurons recorded: acoustic feature cells n = 104, 18.9%, monkey identity cells n = 27, 4.9%, call cells n = 37, 6.7% (among them 25 cells i.e. 4.5% were purely call selective, namely non-significant to identity and acoustic features); Monkey M_b_ had 468 neurons recorded: acoustic feature cells n = 98, 20.9%, monkey identity cells n = 29, 6.2%, call cells n = 41, 8.8% (among them 21 cells i.e. 4.6% were purely call selective, namely non-significant to identity and acoustic features). To visualize the neural representation of each sub-group of neurons on low dimensions, MDS (Multi-Dimensional Scaling) was used. It calculated the distances (Euclidean distance) between the sample points on the high dimensional space, and projected the samples on a 2- or 3- low dimensional space.

Both synthetic dye (Cal-520AM) and GCaMP have been shown to have relatively fast rising kinetic rising kinetic (e.g. ~60 ms for Cal-520AM; ~150 ms for GCaMP6f) for spike detection, in reflecting the presence of individual spikes as well as the total number of spikes within spike trains [10-11]. Thus, the integrated Ca2+ signal used in our call-specificity analysis should reflect the total number of spikes evoked by the call.

**Call Selectivity Index (CSI)** was calculated based on the equation CSI = (R_pref_-R_nonpref_)/ (R_pref_+R_nonpref_), where R_pref_ and R_nonpref_ represent the mean ΔF/F evoked by the preferred call and all other calls, respectively. The **Modulation Index (MI)** was calculated based on peak ΔF/F values by the equations below (Phee cell as an example): MI = (R_TwP_-R_P_)/ (R_TwP_+R_P_), where R_TwP_ and R_p_ represent the average peak ΔF/F responses evoked by TwitterPhee and Phee, respectively. To analyze the nearest-neighbor distances for all call-selective cells regardless of call selectivity, a bootstrap sampling method was used. For each trial of sampling, the same number of neurons as in each call-selective neuronal population was randomly selected from the call-selective populations in each imaging field. We used 500 sampling trials and obtained an averaged cumulative percentage plot of nearest-neighbor distances.

**Code and data availability.** Custom MATLAB scripts used to analyze and plot all data, and original data collected in this study are available from the corresponding authors upon request.

**References**

1. Zeng, HH, Huang, JF, Chen, M*, et al.* Local homogeneity of tonotopic organization in the primary auditory cortex of marmosets. *Proc Natl Acad Sci U S A*. 2019; **116**(8): 3239-44.

2. O'Herron, P, Shen, Z, Lu, Z*, et al.* Targeted labeling of neurons in a specific functional micro-domain of the neocortex by combining intrinsic signal and two-photon imaging. *J Vis Exp*. 2012(70): e50025.

3. Sadakane, O, Masamizu, Y, Watakabe, A*, et al.* Long-Term Two-Photon Calcium Imaging of Neuronal Populations with Subcellular Resolution in Adult Non-human Primates. *Cell Rep*. 2015; **13**(9): 1989-99.

4. Takahashi, DY, Fenley, AR, Teramoto, Y*, et al.* LANGUAGE DEVELOPMENT. The developmental dynamics of marmoset monkey vocal production. *Science*. 2015; **349**(6249): 734-8.

5. Tada, M, Takeuchi, A, Hashizume, M*, et al.* A highly sensitive fluorescent indicator dye for calcium imaging of neural activity in vitro and in vivo. *Eur J Neurosci*. 2014; **39**(11): 1720-8.

6. Chen, TW, Wardill, TJ, Sun, Y*, et al.* Ultrasensitive fluorescent proteins for imaging neuronal activity. *Nature*. 2013; **499**(7458): 295-300.

7. Nimmerjahn, A, Kirchhoff, F, Kerr, JN*, et al.* Sulforhodamine 101 as a specific marker of astroglia in the neocortex in vivo. *Nat Methods*. 2004; **1**(1): 31-7.

8. Kerlin, AM, Andermann, ML, Berezovskii, VK*, et al.* Broadly tuned response properties of diverse inhibitory neuron subtypes in mouse visual cortex. *Neuron*. 2010; **67**(5): 858-71.

9. Ohki, K, Chung, S, Ch'ng, YH, et al. Functional imaging with cellular resolution reveals precise micro-architecture in visual cortex. Nature. 2005; **433**(7026): 597-603.

10. Chen, TW, Wardill, TJ, Sun, Y, et al. Ultrasensitive fluorescent proteins for imaging neuronal activity. Nature. 2013; **499**(7458): 295-300.

11. Tada, M, Takeuchi, A, Hashizume, M, et al. A highly sensitive fluorescent indicator dye for calcium imaging of neural activity in vitro and in vivo. Eur J Neurosci. 2014; **39**(11): 1720-8.
